# As the Egg Turns: Monitoring Egg Attendance Behavior in Wild Birds Using Novel Data Logging Technology

**DOI:** 10.1371/journal.pone.0097898

**Published:** 2014-06-02

**Authors:** Scott A. Shaffer, Corey A. Clatterbuck, Emma C. Kelsey, Alex D. Naiman, Lindsay C. Young, Eric A. VanderWerf, Pete Warzybok, Russell Bradley, Jaime Jahncke, Geoff C. Bower

**Affiliations:** 1 San José State University, Department of Biological Sciences, San Jose, California, United States of America; 2 Stanford University, Department of Aeronautics and Astronautics, Stanford, California, United States of America; 3 Pacific Rim Conservation, Honolulu, Hawaii, United States of America; 4 Point Blue Conservation Science, Petaluma, California, United States of America; Institut Pluridisciplinaire Hubert Curien, France

## Abstract

Egg turning is unique to birds and critical for embryonic development in most avian species. Technology that can measure changes in egg orientation and temperature at fine temporal scales (1 Hz) was neither readily available nor small enough to fit into artificial eggs until recently. Here we show the utility of novel miniature data loggers equipped with 3-axis (i.e., triaxial) accelerometers, magnetometers, and a temperature thermistor to study egg turning behavior in free-ranging birds. Artificial eggs containing egg loggers were deployed in the nests of three seabird species for 1–7 days of continuous monitoring. These species (1) turned their eggs more frequently (up to 6.5 turns h^−1^) than previously reported for other species, but angular changes were often small (1–10° most common), (2) displayed similar mean turning rates (*ca*. 2 turns h^−1^) despite major differences in reproductive ecology, and (3) demonstrated distinct diurnal cycling in egg temperatures that varied between 1.4 and 2.4°C. These novel egg loggers revealed high-resolution, three-dimensional egg turning behavior heretofore never measured in wild birds. This new form of biotechnology has broad applicability for addressing fundamental questions in avian breeding ecology, life history, and development, and can be used as a tool to monitor birds that are sensitive to disturbance while breeding.

## Introduction

The vast majority of bird species exhibit disparate incubation behaviors from their Theropod ancestors, most markedly in their parental physical contact with a clutch of eggs [1]–[4]. Contact incubation likely provided a variety of evolutionary advantages for birds, including a shortened incubation period, accelerated rate of embryonic development, and greater control of incubation conditions [2]. Whereas dinosaurs and reptiles buried eggs, most extant bird species must guard and incubate eggs until hatching. While egg turning behavior has not been studied as extensively as parental attendance patterns, egg physiology, or hatching success, a number of studies have revealed that a lack of egg turning can retard the utilization of albumen by the embryo, resulting in abnormal chick development and reduced hatching success [5], [6]. Incubation temperatures are also critical for embryonic development, hatching success, and sometimes offspring phenotype [1], [7]–[9]. The poultry industry uses knowledge of optimal egg turning rates, magnitude of angle changes, humidity, and temperatures to maximize hatchability of domestic fowl [6], [10]–[13]. However, these parameters are more difficult to quantify in wild birds. Previous studies have examined egg turning in wild birds by using nest observations [14], marked eggs [15], [16], video ethograms [17], [18], telemetric eggs with attitude and temperature sensors [19]–[21], and data loggers with biaxial (i.e., two-dimensional) accelerometers and temperature thermistors placed in artificial eggs [22]–[24]. The technological advancements of the latter studies allowed for more precise analysis of egg turning and incubation temperatures. However, attitude sensors [19]–[21] or biaxial accelerometers [22]–[24] cannot resolve dynamic changes in three-dimensional egg orientation (i.e., roll, pitch, and yaw) or measure true angle changes during rotation events without assistance of a magnetometer, thus limiting a full characterization of egg turning behavior in birds.

New microtechnologies incorporated into smart phones and tablet computers (e.g., 3-axis accelerometers and magnetometers) have facilitated a variety of studies that examine body orientation and characterize movements of free-ranging animals [25]–[28]. Until now, the full potential of this technology to investigate egg turning behavior has not been realized. Here, we use novel egg loggers that record accurate three-dimensional orientation (by combining 3-axis accelerometers and magnetometers) and temperature every second to closely examine egg turning rates in wild birds. We deployed our egg loggers for 1–7 days under incubating Cassin's auklets (*Ptychoramphus aleuticus*), western gulls (*Larus occidentalis*), and Laysan albatrosses (*Phoebastria immutabilis*). These species represent a wide range in body masses (250–3000 g), egg masses (27–300 g), and clutch sizes (1–3 eggs), see Table S1 in File S1. Moreover, they nest in different ecosystems (temperate vs. tropical), show significant variation in nesting habitat types (burrow vs. surface nesting), and differ in temporal parental turnover rates (nightly vs. every 30 days). Consequently, we hypothesized that egg turning rates and activity patterns would vary among species as a result of differences in breeding ecology. However, given the oblong shape of eggs from all three species, we predicted that all three species would turn the egg most frequently about the roll axis. Furthermore, we hypothesized that egg temperatures would be influenced by ambient temperature fluctuations and that these changes could affect egg turning rates.

## Methods and Materials

### Ethics Statement

All animal research was conducted in accordance with approvals from San Jose State University Institutional Animal Care and Use Committee (SJSU 978, 980), Pacific Rim Conservation, Point Blue Conservation Science, California Parks, and the UC Natural Reserve System. Bird Banding permits, Migratory Bird Treaty Act permits, and Special Use Permits for all research were granted by the US Fish and Wildlife Service, US Geological Survey, and the State of Hawaii.

### Egg Logger Deployment

Pilot tests of loggers housed in artificial eggs were performed prior to conducting field deployments. Temperature measurement accuracy and battery life of the egg loggers were tested and verified using a standard poultry incubator with automatic egg turner (Top Hatch Incubator, Brower Equipment, Houghton, IA, USA), in which egg ambient temperature (34°C±6°C, 55% humidity) and rotation were monitored using visual observations (1–3 times daily) for periods of 24 to 240 hours. Changes in egg logger orientation were tested and verified by comparing a video recording of an egg being turned manually with an animation created by post-processing the data collected by the egg logger.

Egg loggers were deployed in the nests of each species for 2–12 days in 2012 (Table 1). Study nests were chosen based on accessibility with minimal disturbance to the bird and the rest of the colony. The majority of Cassin's auklet nests used in this study were in artificial nest boxes used by Point Blue Conservation Science for regular monitoring. During the deployment of egg loggers in auklet nests, the natural auklet egg was removed, placed into in a poultry incubator for the length of the deployment, and returned to the original nest when the egg logger was removed. Because western gulls naturally have multi-egg clutches, it was not necessary to place real eggs in an incubator. Rather, clutch sizes of all study nests were either enlarged from two to three eggs by adding an egg logger or maintained at three eggs by adding an egg logger and transferring a real egg to the nest of another gull temporarily. All albatross eggs were candled 10–14 days post lay and only those nests with infertile eggs were studied. Infertile eggs were removed and replaced with an egg logger, and the infertile eggs were collected for contaminants sampling. Upon completion of each deployment, egg loggers were simply removed and the nest remained empty, thereby encouraging the albatrosses to abandon their breeding attempt and not waste energy on eggs that would never hatch. Study locations and nest characteristics are given in Table S1 in File S1.

**Table 1:** Deployment information for study species.

Species	Deployment		Loggers		
	Dates	Duration (d)	Deployed	Data Used	
Cassin's auklets	April-July 2012	3.51±2.67	35	26	
Western gulls	May-June 2012	4.51±2.53	17	17	
Laysan albatrosses	Dec 2012-Jan 2013	6.15±1.63	19	17	

Data from egg loggers were excluded from analyses if deployments were too short (<24 hours), a bird abandoned the nest, or the data logger malfunctioned.

### Egg Logger Design

Data loggers containing 3-axis accelerometers and magnetometers capable of sensing 1–2° angular changes in roll, pitch, and yaw were housed in replicate eggs (size, shape, and approximate mass; Figure S1 and File S1) of Cassin's auklets, western gulls, and Laysan albatrosses. Egg orientation was estimated by measuring the direction of two non-parallel, inertially-fixed vectors: acceleration due to gravity and the Earth's magnetic field. Low-cost, 3-axis micro-electro-mechanical systems (MEMs) accelerometers and magnetometers capable of sensing these vectors were used.

In addition to measuring acceleration due to gravity, the 3-axis accelerometers also registered motion (accelerations) of the sensor. The dynamics associated with these movements were filtered out in post-processing, limiting the recorded orientation measurements to those times when the egg was stationary. Therefore, it was possible to accurately resolve the orientation before and after the egg was rotated, but with decreased accuracy during a rotation event.

The 3-axis magnetometer measured the direction of the local magnetic field strength, which is a combination of the Earth's magnetic field and any nearby disturbances. Magnetic disturbances due to the egg itself (hard and soft iron effects) were calibrated during post-processing (see File S1). External disturbances could bias the absolute heading (yaw) estimate; however, we were interested primarily in the relative egg yaw angle with respect to its initial orientation. It should be noted that the magnetometer was only required for heading measurements, as the accelerometer alone is sufficient for determining steady state roll and pitch angles. However, the magnetometer aids in tracking orientation during dynamic motions of the egg as it is not biased by movement.

Sensors selected for the loggers were contained in ST Microelectronics LSM303DLHC, a single chip with an integrated 3-axis accelerometer (1e-4 m/s^2^ resolution), 3-axis magnetometer (0.2 µT resolution), and a temperature thermistor (0.125°C resolution, <2°C accuracy). Given that loggers were centered inside the eggs, temperature readings were considered to be core egg temperature and not peripheral temperature. Logger operation was controlled by a Microchip PIC24F32K302 microcontroller, which triggered measurements once per second. Raw sensor measurement data were recorded to a μSD card at a rate of approximately 115 KB per hour. A 60-mAh single cell Lithium Polymer battery with a low-voltage protection circuit powered the loggers. In the field, loggers operated for up to 8 days continuously.

### Data Processing

Data were processed with purpose-built routines in MATLAB (The Mathworks, Natick, MA, USA). Raw accelerometer and magnetometer measurements were converted to 3-2-1 Euler angles (expressed as yaw, pitch, roll; Figure S2) using an Extended Kalman Filter (EKF) to estimate instantaneous egg orientation (see File S1). Orientation changes were distilled into statistics that detected rotation events with a minimum angle change of 10°. This threshold was used 1) for comparability with previous studies [22]–[24] and 2) because it also approximated the point of inflection in the cumulative distribution between turning events and angular change (see File S1). The first 6 hours of all deployments and any periods of abandonment after a trial commenced were excluded from analyses. Egg abandonments were detected by measurements of low egg temperature and lack of egg turning for periods greater than 3–4 hours.

The relatively low resolution of the temperature sensors (± 0.125°) produced jagged step changes in egg temperatures over short temporal periods (Figure S3). Consequently, a smoothing function was applied that created a moving average using 5000 points (seconds) at each step through the temperature measurements. This provided a smoothed temperature profile without a drastic loss of the measured sensor variation.

Because egg loggers recorded continuously for several days, we evaluated whether egg turning rates differed between day and night using ephemeris tables to determine the time of local sunrise and sunset at each colony. Colony latitude and longitude (Table S1 in File S1) were obtained using a handheld GPS or images from Google Earth. Mean egg turning rates for day and night (Table 2) were determined for each individual within a species and paired t-tests were used to test for differences in egg turning rates between day and night. All data were presented as means (± SD) unless otherwise stated and α≤0.05 was used for all statistical tests.

**Table 2:** Comparison of turning frequencies (mean ± SD) during the day and night for each bird species.

	Turns per hour				
Bird species	Day	Night	*t*-stat	*df*	*P*
Cassin's auklets	1.8±0.3	2.6±1.3	−2.6	47	**0.011**
Western gulls	2.1±0.4	2.0±0.5	0.5	27	0.625
Laysan albatrosses	2.1±0.7	2.0±0.4	0.6	37	0.582

## Results and Discussion

We made 71 deployments of artificial eggs with loggers in nests of Cassin's auklets (N = 35), western gulls (N = 17), and Laysan albatrosses (N = 10; with repeated deployments in 5 nests for a total of 19 deployments) for durations averaging 3.5 to 7.2 days (max = 12.0 days; Table 1). All birds initially accepted the artificial eggs, but auklets were more sensitive to the disturbance from nest visitations by researchers and eventual acceptance of the artificial egg logger was lower than in the other two species. Twelve of 35 auklets abandoned their breeding attempt after 1–3 days; however, 50% of these birds laid another egg and hatched a chick later in the same season. Four of 19 albatross deployments resulted in abandonment after 2–4 days but two were the result of the artificial egg breaking open during the deployment. No gulls abandoned their eggs during our study. Subsequent changes in egg design in 2013 resulted in a substantial reduction in abandonments by auklets (Kelsey et al. in prep) and no abandonments in gulls or albatrosses (Clatterbuck et al. in prep).

### Biological Significance of New Egg Loggers

**Figure 1:**
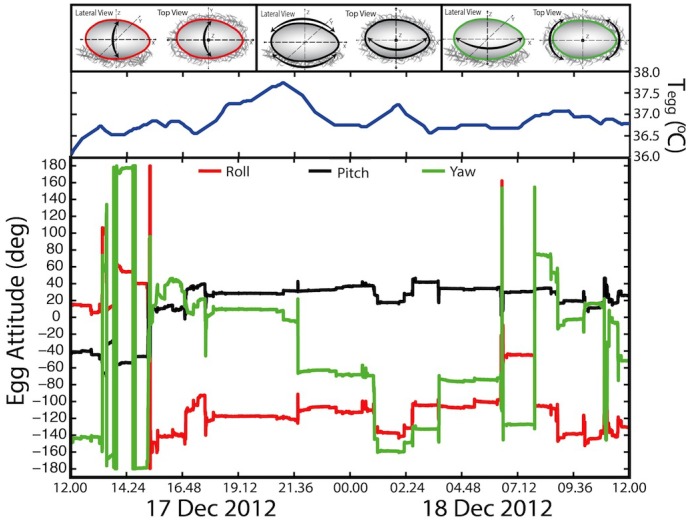
Representative 24-hour time series showing egg attitude and temperature in a Laysan albatross. Shown are the roll, pitch, and yaw angles and the corresponding egg temperature in the pane above plotted against local date and time starting at 12.00 noon. Of note are the large angular changes in yaw attitude compared to the attitudes of roll and pitch. Additional time series are shown in Figure S4.

**Figure 2:**
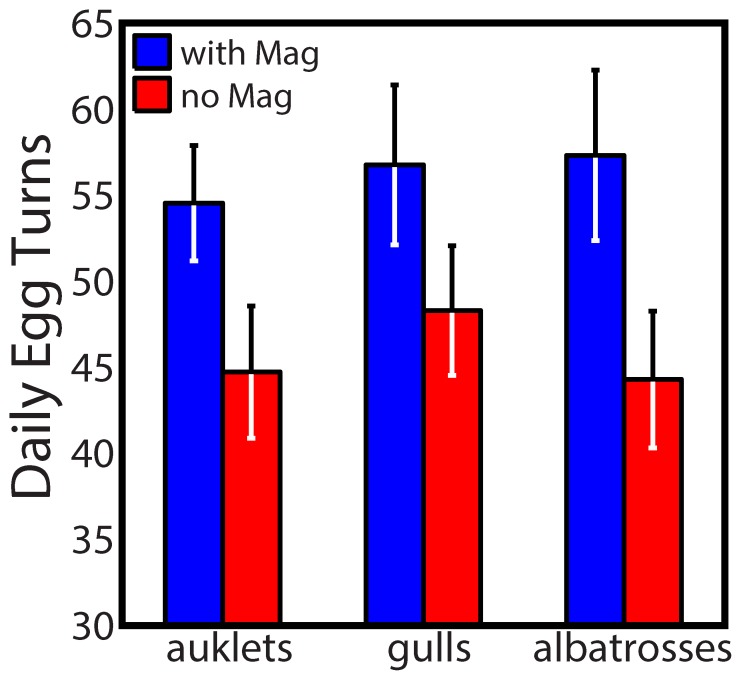
Comparison of daily egg turning rates in auklets, gulls, and albatrosses with and without the use of a magnetometer. Egg turning was measured using egg loggers that had a triaxial accelerometer and magnetometer. However, datasets of ten individuals within each species were compared with and without incorporating the use of the magnetometer data. Shown are means ± SE. Wilcoxon signed-rank tests were used to evaluate statistical differences between turning rates for all individuals within a species. Turning rates were significantly greater for all three species when the magnetometer data were incorporated.

The egg loggers we developed for this study had two major advancements from loggers used previously [22]–[24]. Firstly, our egg loggers had triaxial rather than biaxial accelerometers, allowing resolution of egg turning in three dimensions instead of two. Birds exhibit a variety of movements that can facilitate egg turning, whether intentional or not (e.g., rotating, sitting up, leaning to the side, etc.), and these movements were captured in three dimensions (Figures 1 & S4, and animations online in Files S2, S3, and S4). Secondly, we incorporated a triaxial magnetometer in our loggers that measured egg orientation (i.e., true angular changes) in relation to the Earth's magnetic field. When combined with the accelerometer, our loggers provided a more accurate re-creation of egg turning patterns based on 1) accelerating motion and 2) magnetically-oriented turn angles. To evaluate how this combination improved our estimates of egg turning, we selected ten individuals from each species and compared egg turning behavior using the same dataset with and without magnetometer measurements. The differences in estimated turning rates were significant (Wilcoxon signed-rank test; *W_10_* = 0, *P* = 0.002) for all three species. In each case, egg turning rates were 10–30% higher when magnetometer data were incorporated in the analysis, which equates to 10–20 additional turns of the egg per day (Figure 2) that would potentially be missed by using accelerometers only.

### Comparative Egg Turning Behavior

**Figure 3:**
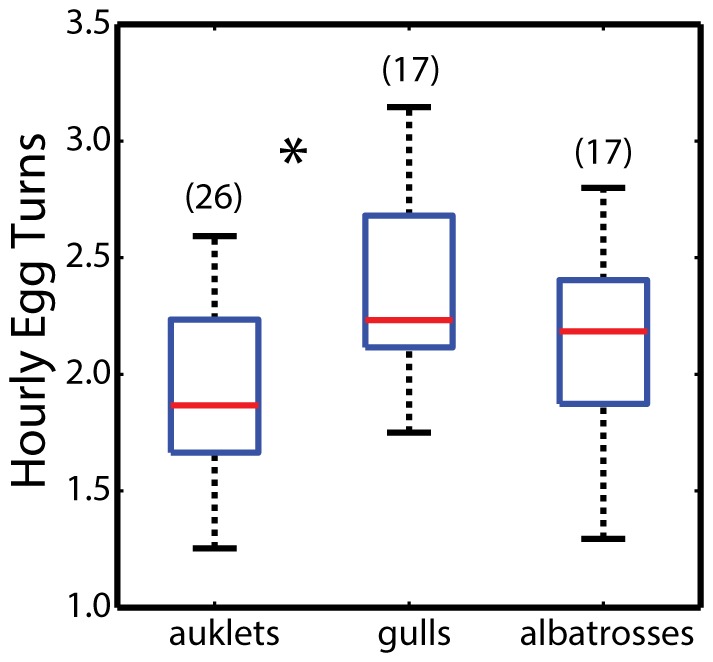
Summary of egg movements in Cassin's auklets, western gulls, and Laysan albatrosses. Shown are box & whisker plots representing the median values (red lines), upper and lower quartiles, upper and lower extremes for all individuals of a particular species, and the dot is an outlier. Sample sizes of individual deployments for each species are enclosed in (). Albatross deployments included a mixture of single deployments (N = 10 nests) and repeated deployments (N = 5) in the same nest. Statistical differences (ANOVA and pairwise comparisons) among species are denoted by asterisks. Turning rates were based on a minimum threshold of 10° of angular change in egg orientation to be counted as part of the rate (see Figure 4 for additional detail).

**Figure 4:**
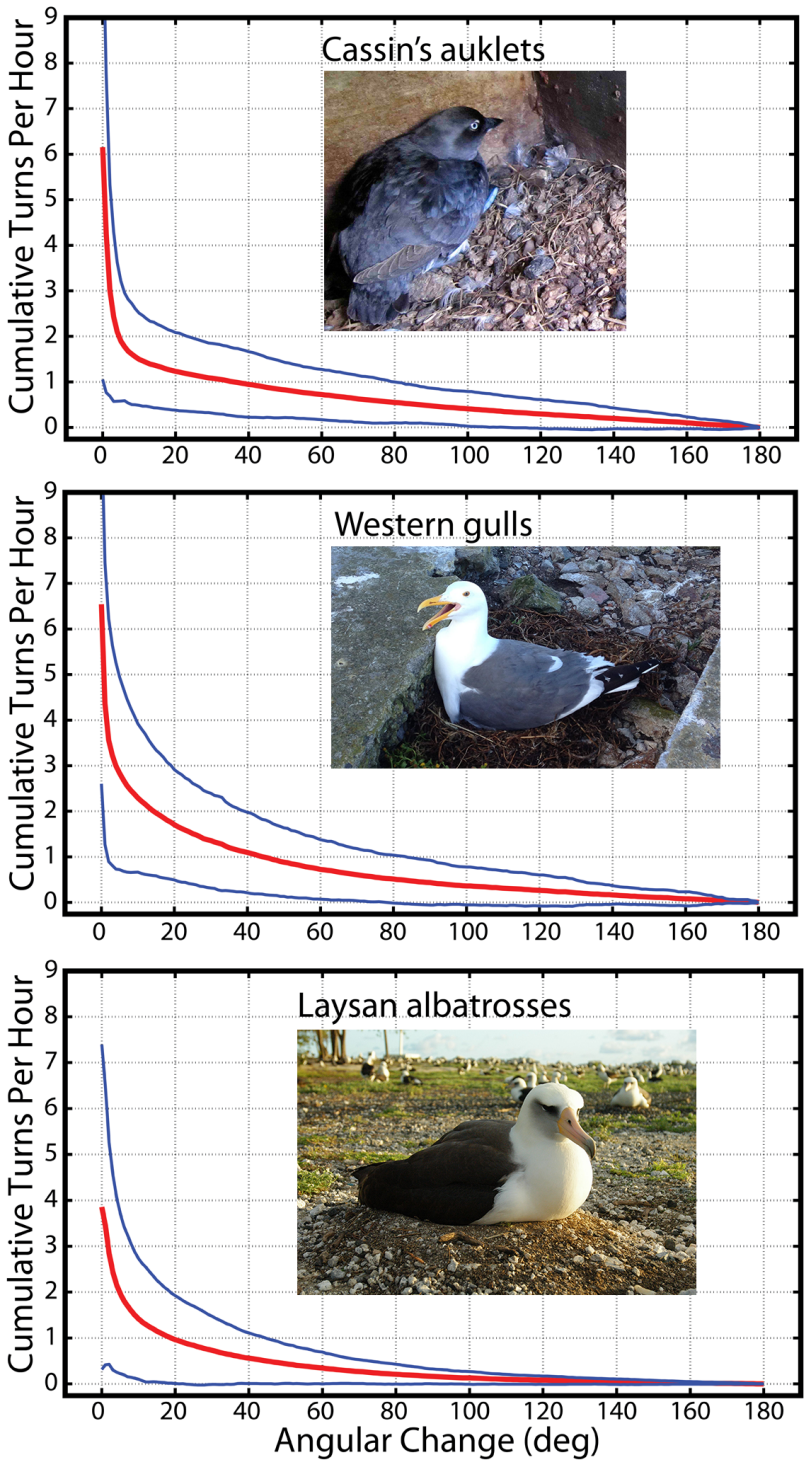
Cumulative turns h^−1^ as a function of turn angle for each species. Plotted are the means (red line) and confidence intervals (blue lines) for each species. These plots show that a preponderance of small changes in egg orientation leads to a high rate of egg turning and vice versa. Angular change is based on the combination of all three attitudes (roll, pitch, and yaw) determined using Euler's theorem (see Figure S2 and File S1). Turning rates shown in Figures 2 & 3 were estimated using a minimum threshold of 10° of angular change to be counted as a turning event. Without using a minimum threshold, turning events ranged between 5–8 turns per hour for each species and these were largely (>95% of all angle changes) based on changes of 1–2°. Photos by ECK and SAS.

Median hourly egg turning rates for all three species varied from 1.8 turns h^−1^ to 2.2 turns h^−1^ (Figure 3). The difference in turning rates among all three species was significant (ANOVA, *F*_2,59_ = 6.52, *P* = 0.003), although multiple comparison tests revealed that turning rates only differed between auklets and gulls (*P* = 0.002). The difference in turning rate between auklets and gulls was likely due to variations in 1) clutch size, because auklets incubate a single egg whereas gulls incubate 1–3 eggs that require greater parental attention, and/or 2) nesting habitat, because auklets nest in burrows that protect against most biological and physical perturbations whereas gulls are surface nesters in large colonies that require greater vigilance from colony disturbances (e.g., aggression between neighbors, egg cannibalism, etc.). Albatrosses, which are surface nesters like gulls, but incubate a single egg like auklets, had turning rates intermediate to auklets and gulls (Figures 3 & 4). Thus, there was variation in turning rates among species as we hypothesized. However, there did not appear to be a general pattern explained by differences in clutch size, egg size, and nesting habitat. This result suggests that egg turning rates may be relatively conserved among seabirds but further research is required, especially among related species that breed in different habitats. For example, one future study we hope to conduct involves comparing egg turning behavior among different albatross species. This comparison could be valuable because albatrosses range from tropical to sub-Antarctic habitats, yet clutch size is fixed at one egg and nesting habits are similar. Such a comparison could potentially shed light on whether environment plays a significant role in egg turning behavior among different bird species.

Comparative studies using similar biotechnology (i.e., biaxial accelerometers only) quantified egg turning rates in Adėlie penguins (*Pygoscelis adeliae*) and reported ranges of 1.4–3.2 turns h^−1^ [22]–[24]. Each study used the same data loggers at the same geographic location (but in different years), yet turning rates were quite variable within the same species. Presently, there is a paucity of research involving multi-year studies on the same species. However, it is conceivable that intraspecific egg attendance patterns could vary seasonally, or that these patterns are weather dependent [23], especially in extreme conditions like the Antarctic. A previous review [2] of egg turning rates for 61 species ranging in size from small passerines to large swans reported that egg turning rates varied from 0–12 turns h^−1^, but some of this variability was likely due to the manner in which egg turning was measured. We believe the egg loggers used in this study can provide a standardized method to study egg attendance patterns that allows accurate inter- and intraspecific comparisons of egg turning rates.

Previous research qualified egg turning only in a general sense (e.g., about the long or short axes [13], [15]) but quantification of rotation about a specific axis is scant. Given the oblong egg shapes of all three species (Figure S1), we predicted that parents would turn the egg along the roll axis (Figures 1 & S4). However, our results showed that the largest angular changes occurred in the yaw angle (Figures 1 & S4, and animations online in Files S2, S3, and S4). Rotation about the yaw axis could induce an egg to roll if an adult bird increased or decreased the pitch angle and the egg rotated without slipping. Gulls and albatrosses shuffle their feet and body frequently while rotating around the nest cup, but we have not observed gulls or albatrosses using their bill to turn an egg, as reported for other species [14]–[16], [18]. Rotating around the nest cup permits egg turning without the parent lifting off the egg, thus minimizing exposure to ambient temperatures and risk of predation. No previous studies we are aware of have described egg rotations and specific orientations for a crevice or burrow-nesting species.

### Diurnal Patterns in Egg Turning and Incubation Temperature

Diurnal egg turning patterns in wild birds are rarely studied because it is difficult to make observations in darkness (but see 16, 18). We were able to examine diurnal incubation behaviors more closely and predicted that egg turning rates would be lower at night for species not generally active in the colony at night. Indeed, our observations confirm that nesting western gulls and Laysan albatrosses were generally quiescent at night whereas Cassin's auklets followed a nocturnal rhythm where partners exchanged nesting duties. In contrast to what we predicted, turning rates were indistinguishable between night and day for gulls and albatrosses (Table 2, see animations online in Files S2, S3, and S4). Egg turning rates in auklets were significantly greater, increasing by 44% during night time periods (Table 2, Figure S4, and animations online in Files S2, S3, and S4). These results demonstrate the importance of continual egg attendance for chick development [2], [5], [11] because parent birds showed little variation in egg attendance patterns throughout the 24-hour cycle during the periods of study.

**Figure 5:**
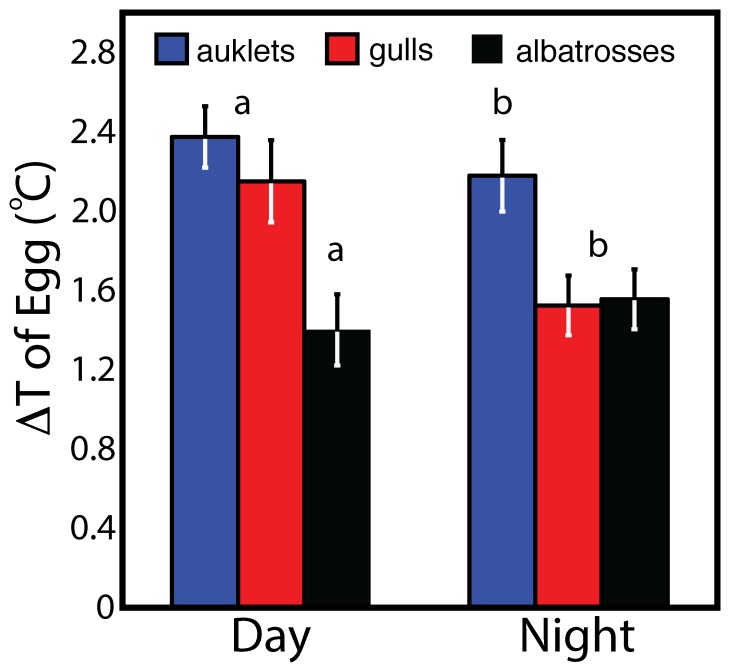
Comparison of Δ (delta) egg temperatures among Cassin's auklets, western gulls, and Laysan albatrosses during the day and night. Delta temperatures represent the difference between the maximum and minimum egg temperature of each day and subsequent night cycle. Shown are the mean ± SE for each species where N = 19, 11, and 14 individual deployments of each species (respectively) during the day and N = 19, 15, and 16 individual deployments of each species (respectively) during the night. Statistical differences (*P*<0.05) were designated by symbols a & b. During the day, Δ temperatures of albatrosses were significantly lower than auklets and gulls (a) whereas during the night, both gulls and albatrosses had significantly lower Δ temperatures than auklets (b). See Table 3 for specific temperatures and Figure 6 for temperature cycling.

**Figure 6:**
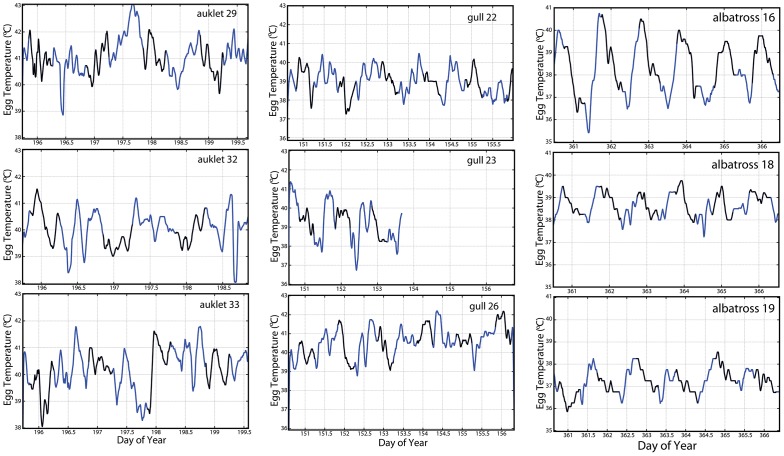
Variations in the cycling of daily egg temperatures of nine individual birds (3 from each species). Each pane shows continuous logging of smoothed egg temperatures across multiple days. Sections of black line overlaid on the blue line denote night time periods based on ephemeris tables for the geographic coordinates of each colony (Table S1 in File S1). Temperature scales are consistent within each species but temporal periods varied slightly between individuals. However, all individuals shown were from equivalent calendar periods within a species to ensure similarity of environmental conditions that could potentially influence incubation behavior. Cycling coincides with a diurnal pattern and there is a distinct trend towards increased rhythmic temperature cycling with increasing egg size (i.e., species size). The pattern is less clear for the auklets but these birds nest in burrows or boxes and may be influenced less by diurnal patterns.

Egg temperatures varied diurnally by as much as 2.4°C on average (Figure 5); however, day time egg temperatures varied more than night time egg temperatures for surface nesting gulls only (Table 3). This temperature variation may be related to general egg attendance patterns in gulls where they 1) care for a larger clutch that requires more turning, 2) experience high levels of colony activity, 3) have frequent nest duty exchanges between partners, or some combination of all three. Despite variations in egg temperature in both day and night, only albatrosses exhibited a clear pattern in temperature cycling that was associated with a diurnal cycle (Figure 6). A similar pattern was observed in large cranes (*Grus* spp.; 21) but the pattern was less evident in the auklets and gulls we studied (Figure 6). Hence, the variation in cyclical pattern could result from the 1) thermal inertia of small vs. large eggs, 2) differences in clutch size [29], [30], 3) behavior and heat input of the adult incubating the egg [31], [32], or 4) simple entrainment of the egg that mirrored diurnal patterns of adult body temperature. Despite variations in egg temperature, there was no discernible relationship between egg temperature and the timing of egg turning for all three species.

**Table 3:** Minimum and maximum day and night egg temperatures of each bird species.

Species		N	Min (°C)	N	Max (°C)	*t-statistic*	*P*
Cassin's auklets	Day	11	36.7±1.1	18	38.8±1.6	−3.8	<0.001
	Night	14	37.2±1.3	23	38.3±1.5	−2.2	0.031
Western gulls	Day	10	38.2±1.5	11	40.2±1.3	−3.3	0.004
	Night	15	38.4±1.2	15	39.8±1.4	−3.0	0.005
Laysan albatrosses	Day	15	37.2±0.9	13	38.6±1.2	−3.9	<0.001
	Night	16	37.3±0.8	16	38.8±1.0	−4.3	<0.001

Shown are the mean ± SD min and max egg temperatures for each species.

## Future studies

The technological advancements of our egg loggers reveal a wealth of basic information about incubation behavior in wild birds. However, we believe their real value will be demonstrated in future studies with an applied focus. For example, hatching success is a key determinant of overall breeding success in some species [33], [34], and the loggers could be used to investigate whether turning rates and incubation temperatures are influenced by 1) age and experience of the parents, 2) latitudinal variations in nesting habitats (tropical, temperate, and polar), 3) variations in nest construction (or lack of), 4) change across the incubation period [2], [5], [22], or 5) variations that occur with hormone (sensu [23], [24]) or contaminant levels.

Egg loggers have also been used to investigate the effect of disturbance on adult birds that are incubating egg(s) on the nest [22], [35]–[40]. However, we are unaware of any study using egg loggers to evaluate disturbance effects on the egg itself. Many bird species quickly flush off the nest when disturbed, thus kicking the egg and leaving it exposed to cooler temperatures. Although it is less clear how this would affect the developing chick, quantifying the impact to the egg (e.g., additional turns, cooling rates, etc.) is a starting point that could be followed up with developmental studies on embryos. Such physical impacts could be captured with high-resolution loggers like ours that sample every second (or higher). Continued improvements in technology will enhance the capability of these loggers for broader applications in the future.

## Supporting Information

Figure S1 **Logger and its installed orientation next to the replica eggs for each species.** The logger axes and egg axes are labeled for each egg. Axis label at the intersection indicates the axis extending in the third dimension to complete a right handed coordinate system.
(TIF)

Figure S2 **Visual example of the egg orientation described by 3-2-1 Euler angles, as measured by 3-axis accelerometers and magnetometers placed in artificial eggs and deployed in the nests of wild birds.** The egg orientation is achieved by first rotating from North by the yaw angle about the Earth's fixed z-axis (down), followed by rotating by the pitch angle about this intermediate frame's y-axis, and finally rotating by the roll angle about the next intermediate frame's x-axis.
(TIF)

Figure S3 **Example of egg temperature smoothing.** Egg temperature was recorded every second at a resolution of 0.125°C (blue line). For all subsequent analyses, a smoothing function (i.e. moving average with window size of 5000) was applied to reduce the coarseness of the data. Further details are described in File S1. Shown are approximately two days of egg temperature measurements from a Laysan albatross (bird 18).
(TIF)

Figure S4 **Representative 24-hour time series showing egg attitude and temperature in a) Cassin's auklet and b) western gull.** Shown are the roll, pitch, and yaw Euler angles and corresponding egg temperature in the pane above plotted against local date and time starting at 12.00 noon. Of note is the high turning activity for the Cassin's auklet around 20.30 or dusk, when the partners of incubating birds return to the colony each night to exchange incubating duties.
(TIF)

File S1 **Supplemental methods**. Additional methods, data analysis and processing of egg logger data.
(DOCX)

File S2 **Animation of egg turning in a Cassin's auklet.**
(MP4)

File S3 **Animation of egg turning in a western gull.**
(MP4)

File S4 **Animation of egg turning in a Laysan albatross.**
(MP4)
