# Supplementary figures and images for: As the Egg Turns: Monitoring Egg Attendance Behavior in Wild Birds Using Novel Data Logging Technology
Source: PLoS One. 2014 Jun 2;9(6):e97898. doi: 10.1371/journal.pone.0097898 (PMC4041652; doi:10.1371/journal.pone.0097898)

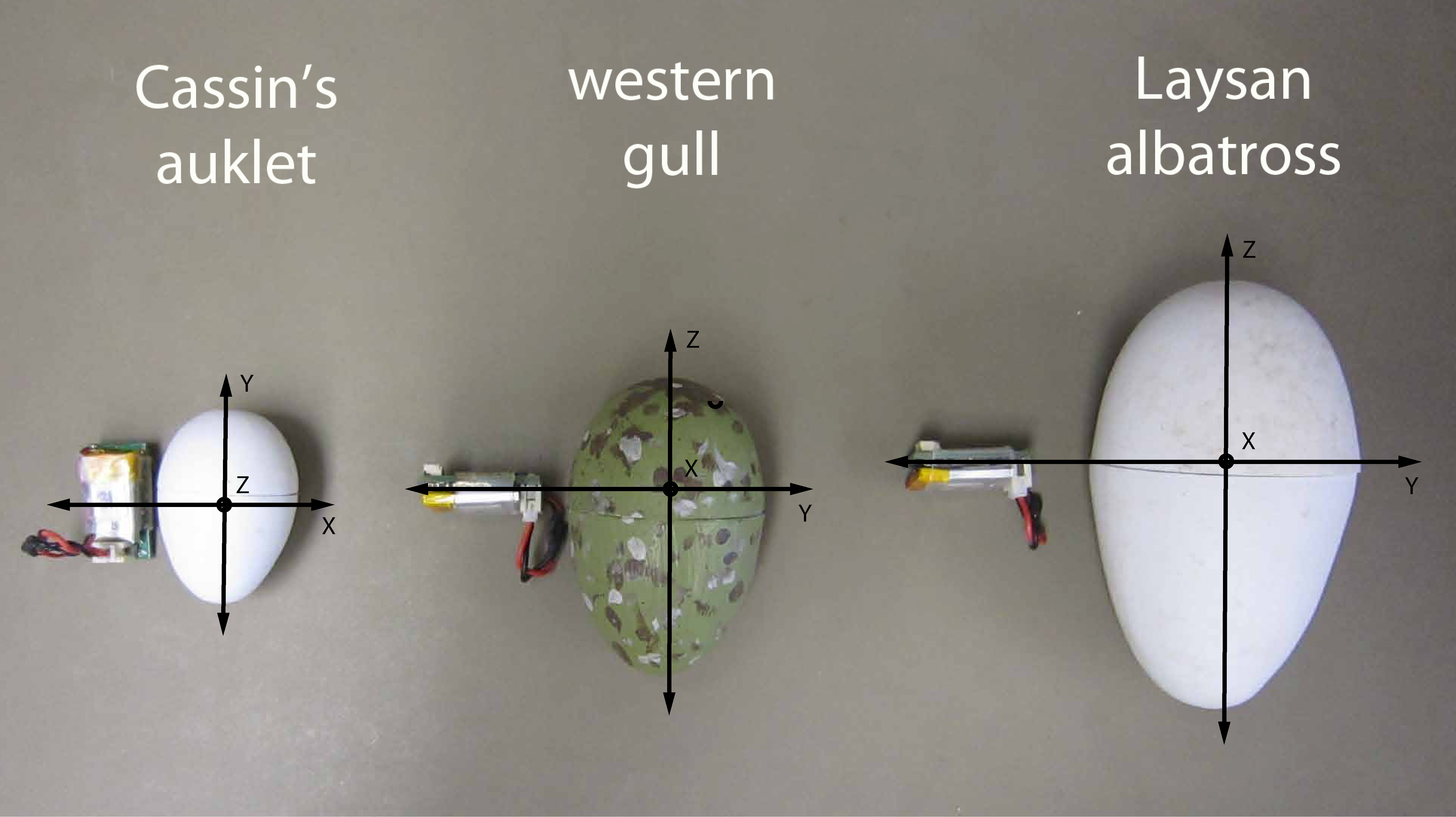

Supplement: Figure S1 — Logger and its installed orientation next to the replica eggs for each species. The logger axes and egg axes are labeled for each egg. Axis label at the intersection indicates the axis extending in the third dimension to complete a right handed coordinate system. (TIF) [file pone.0097898.s001.tif]

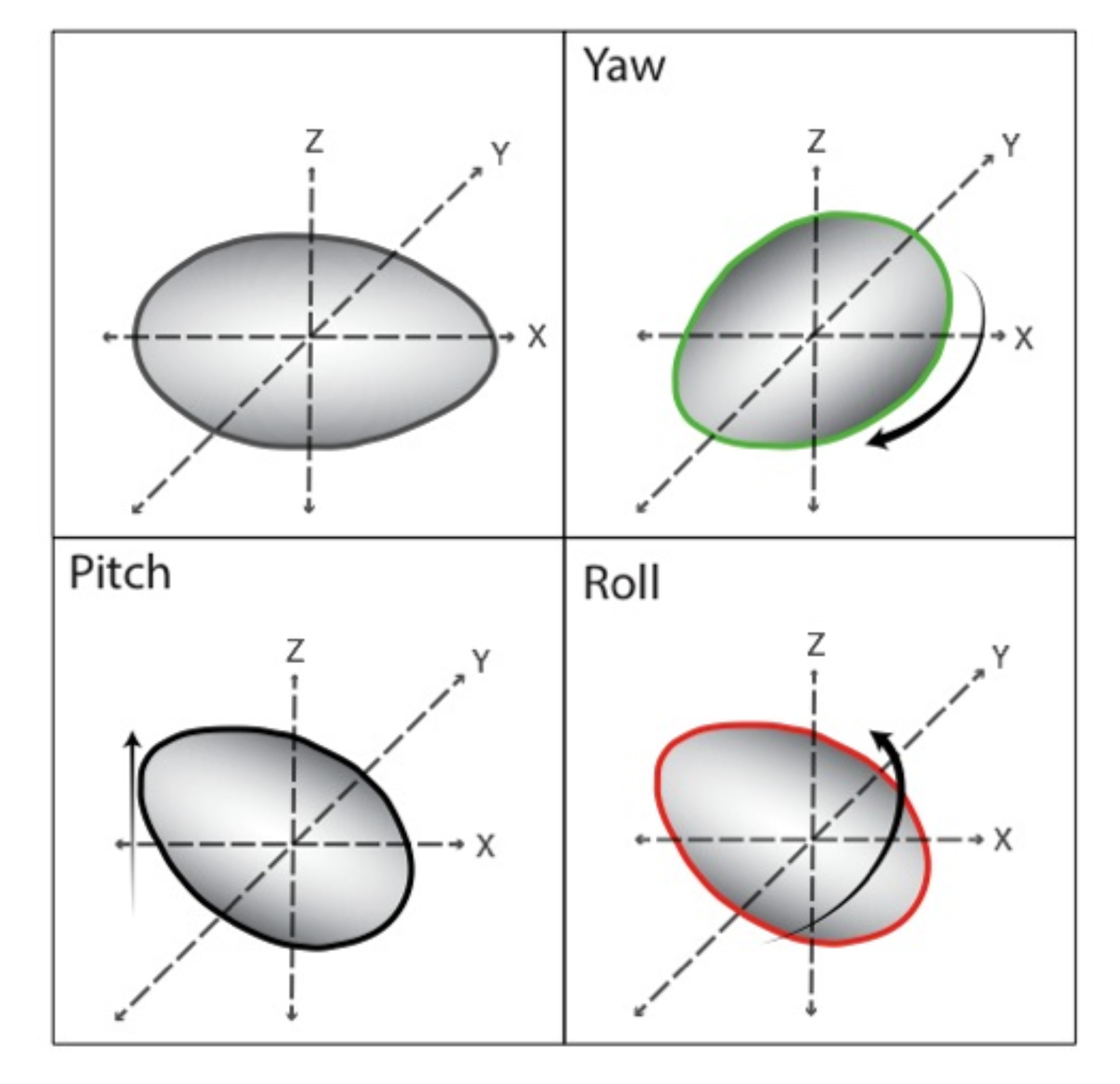

Supplement: Figure S2 — Visual example of the egg orientation described by 3-2-1 Euler angles, as measured by 3-axis accelerometers and magnetometers placed in artificial eggs and deployed in the nests of wild birds. The egg orientation is achieved by first rotating from North by the yaw angle about the Earth's fixed z-axis (down), followed by rotating by the pitch angle about this intermediate frame's y-axis, and finally rotating by the roll angle about the next intermediate frame's x-axis. (TIF) [file pone.0097898.s002.tif]

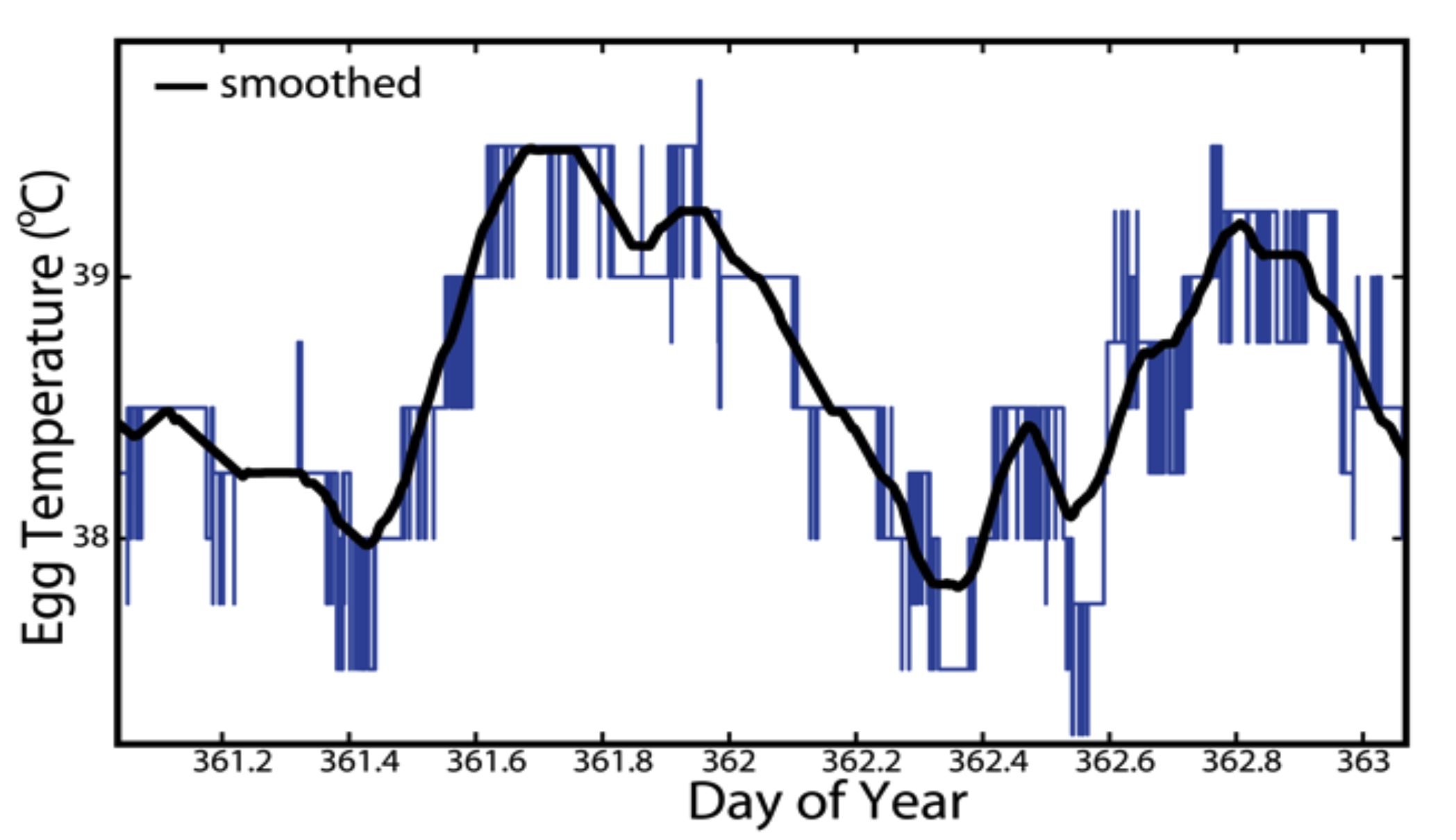

Supplement: Figure S3 — Example of egg temperature smoothing. Egg temperature was recorded every second at a resolution of 0.125°C (blue line). For all subsequent analyses, a smoothing function (i.e. moving average with window size of 5000) was applied to reduce the coarseness of the data. Further details are described in File S1. Shown are approximately two days of egg temperature measurements from a Laysan albatross (bird 18). (TIF) [file pone.0097898.s003.tif]

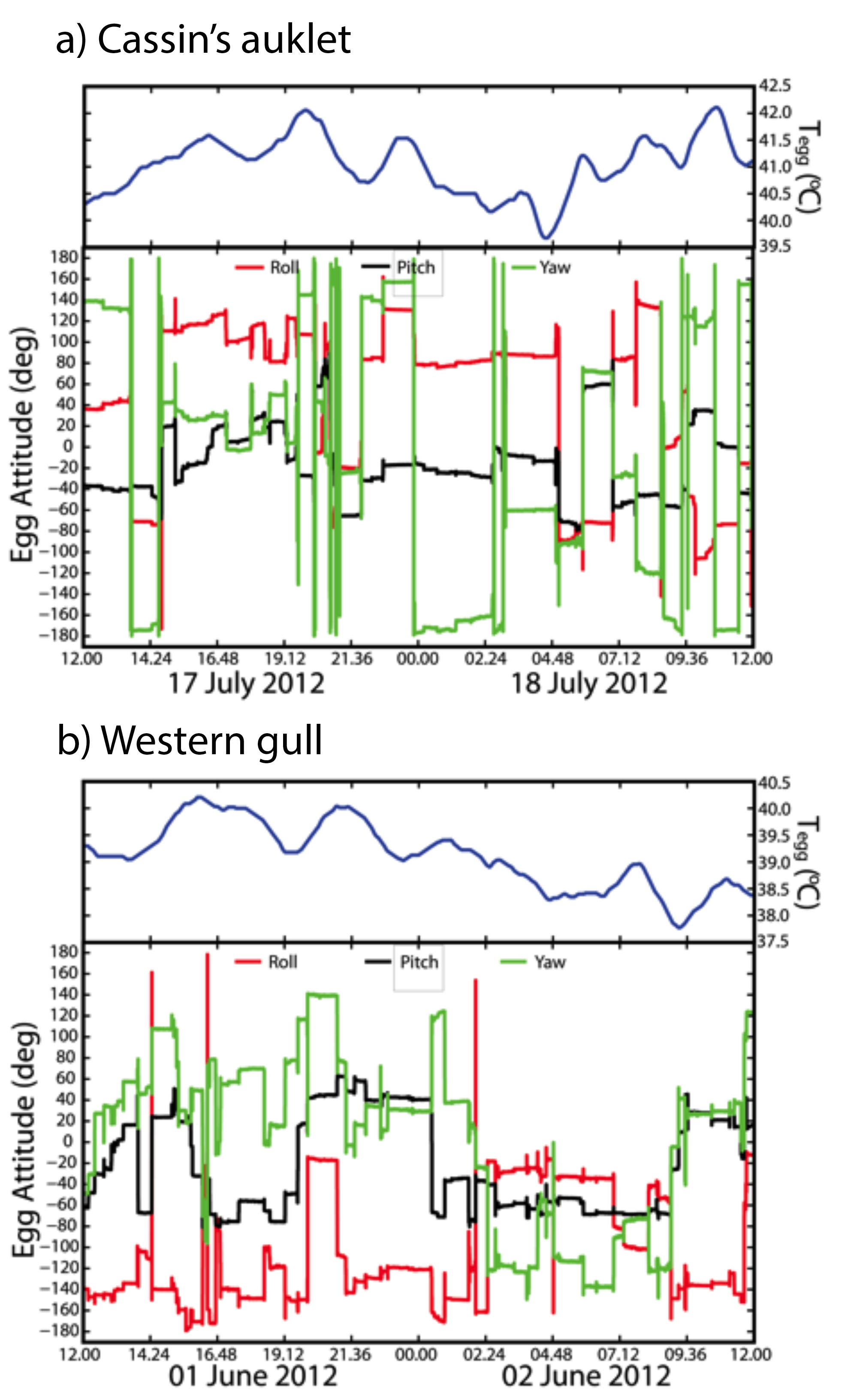

Supplement: Figure S4 — Representative 24-hour time series showing egg attitude and temperature in a) Cassin's auklet and b) western gull. Shown are the roll, pitch, and yaw Euler angles and corresponding egg temperature in the pane above plotted against local date and time starting at 12.00 noon. Of note is the high turning activity for the Cassin's auklet around 20.30 or dusk, when the partners of incubating birds return to the colony each night to exchange incubating duties. (TIF) [file pone.0097898.s004.tif]
