# Supplementary material for: As the Egg Turns: Monitoring Egg Attendance Behavior in Wild Birds Using Novel Data Logging Technology
Source: PLoS One. 2014 Jun 2;9(6):e97898. doi: 10.1371/journal.pone.0097898 (PMC4041652; doi:10.1371/journal.pone.0097898)
Supplement: File S1 — Supplemental methods. Additional methods, data analysis and processing of egg logger data. (DOCX) [file pone.0097898.s005.docx]

**Supplementary Methods**

*Artificial Egg Design* - In addition to meeting the sensor requirements, loggers had to be small and light enough to fit inside the eggs of the three species studied (Table S1 below). For scale, Figure S1 shows a logger next to an artificial egg for each species. Due to size constraints, logger orientation within an egg was different for auklets compared to gulls and albatrosses where the x-axis of the logger was parallel to the long axis of auklet eggs, but for gull and albatross eggs, the x-axis of the logger was perpendicular to the long axis of the egg. An adjustment for this difference in axis orientation was made during data post-processing.

Artificial eggs were designed and created by students of Art and Industrial Design Departments at San Jose State University, San Jose, California. Artificial eggs were made of polystyrene plastic (3 mm thick; SABIC’s Innovative Plastics, Pittsfield, MA) vacuum formed to the approximate size and shape of the natural eggs for each species (Figure S1). Artificial eggs had a twisting lock mechanism to keep both halves together without using adhesive during deployments. However, on 3 occasions, the locking mechanism failed and eggs came apart while a bird was incubating the artificial egg. These logger data were either excluded entirely or retained to the point where it was obvious from temperature recordings (prolonged and sustained temperatures below 30°C), that an egg was no longer being tended. Artificial eggs were filled with a combination of foam rubber and/or thermally neutral wire pulling lubricant (Clear Glide, Ideal Industries Inc.) distributed evenly around the logger within the egg. This gave the artificial eggs mass similar to that of real eggs. However, due to natural variability in egg mass and size, some artificial eggs were 5-30% lighter than the original egg because all egg loggers were activated and packaged in artificial eggs prior to removal of the original egg in a nest, and it was not always possible to account for extreme variations in egg mass or size prior to a deployment.

**Table S1.** Colony and nest characteristics for each bird species. Colony locations included Southeast Farallon Island, CA for auklets, Año Nuevo Island, CA for gulls and Kaena Point, Oahu, HI for albatrosses.

| **Species** | **Colony Location** | **Habitat Type** | **Nest Type** | **Clutch Size** | **N** | **Egg Mass (g)** | **Egg Length (mm)** | **Egg Width (mm)** |
| --- | --- | --- | --- | --- | --- | --- | --- | --- |
| Cassin’s auklets* | 37.42°N, 122.68°W | Temperate | Burrow | 1 egg | 43 | 28.2 ± 2^41^ | 45.6 ± 1.6 | 33.3 ± 1.1 |
| Western gulls* | 37.11°N, 122.33°W | Temperate | Surface | 1-3 eggs | 27 | 76 ± 3.4 | 70.05 ± 1.4 | 46.8 ± 0.5 |
| Laysan albatrosses# | 21.34°N, 158.16°W | Tropical | Surface | 1 egg | 10 | 270 ± 12 | 108.0 ± 4.3 | 67.0 ± 1.8 |

*** - Order Charadriiformes, # - Procellariiformes, 41 - Astheimer, L.B (1986) Egg formation in Cassin’s auklet. *Auk* 103, 682–693 .**

*Determining Orientation from Raw Sensor Data* - This section details the post-processing steps that take the raw accelerometer and magnetometer measurements and convert them into estimates of the egg orientation (expressed as 3-2-1 Euler angles [yaw, pitch, roll], or quaternions, or a rotation matrix). Sensors were calibrated by removing any axis-misalignment, scale factor, or bias effects on the accelerometer, and hard and soft iron effects on the magnetometer. Calibrated sensor measurements were then used in an Extended Kalman Filter (EKF) to estimate the egg orientation. Finally, orientation data were distilled into statistics about the detected egg rotation events.

*Calibration* - For accelerometer and magnetometer sensors, calibration reduced each measurement to a unit vector pointing in the direction of gravity and the local magnetic field, respectively. Due to axis mis-alignment, non-orthogonality, scale factor, and bias errors, as the egg is moved through different orientations, the body x, y, and z axis measurements trace out the surface of an ellipsoid. When properly calibrated the norm of each measurement lies on the surfaces of a sphere of radius 1 (or in engineering units, a sphere of radius 1g = 9.8 m/s^2^, or equal to the local magnetic field strength, which depends on latitude, longitude, altitude, and time of year [42].

An iterative non-linear least squares fitting technique was used [43,44]. First, a subset of the data was selected to use in the calibration. As biases on the accelerometers are typically small, a threshold on the total acceleration magnitude of 0.025 g was used to filter for data that was expected to be stationary. The data used for calibration was then uniformly selected from the remaining subset of data that fell between 10% and 90% of the total time the logger was recording in order to ignore disturbances related to the temperature and magnetic field as the loggers were introduced or removed from the nest. Typically, 500 data points were used in the calibration.

At each iteration, *k*, of the algorithm, a non-linear least squares solution was found for A and B in equation 1.

(1)
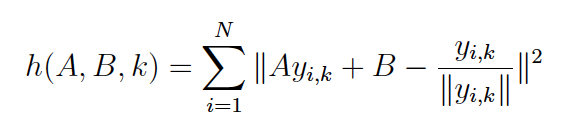


where *y_i,k_* is the measurement vector at time *i* and iteration *k*,
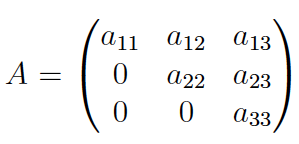
 and
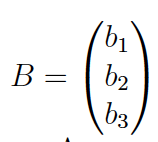


with the *a*'s and *b*'s representing the nine calibration parameters being fit.

An example set of magnetometer data before and after calibration is shown in Figure S5 below. Note the significant offset and distortion in the raw data that is corrected by the algorithm to bring the data to the unit sphere. After the calibrations were performed, checks were made to estimate quality of the calibration on the complete data set and to establish the relative magnitude of the z-component of the magnetic field. First, the mean and standard deviation of the norm of all the calibrated measurements were computed. This metric was typically not as great for the accelerometer because it includes measurements at the time the egg is moving and experiencing more than just the acceleration due to gravity. In most cases, the mean of all the accelerometer readings was within 1% of 1g and the standard deviation was less than 3%. For the magnetometer, the mean was typically within 0.5% of the calibrated field strength and the standard deviation was less than 2% of the calibrated field strength. These limits may be exceeded for short data sets where there was not sufficient coverage of the unit sphere (a wide variety of orientations) represented in the data set.


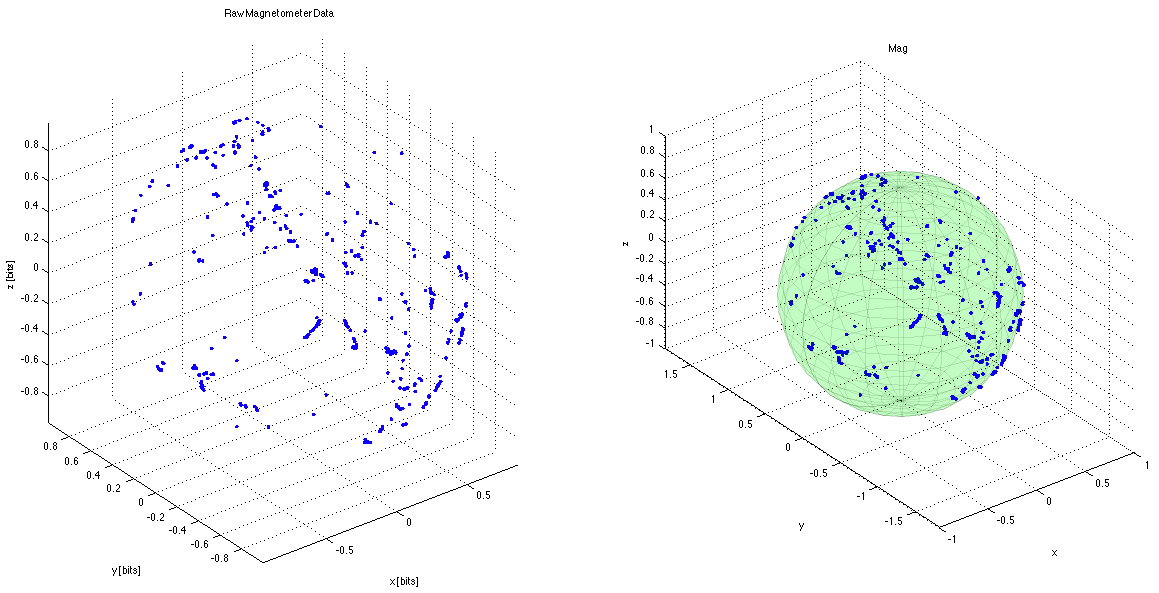


**Figure S5.** **Magnetometer data before (left) and after (right) calibration**.

The second check computed the dot product between the calibrated magnetic field and gravity vectors for the calibration data set. The vertical normalized magnetic field components returned by the dot product were used in an EKF. No attempts were made to calibrate the effect of temperature on the sensors. From recorded temperature data, we expected only small variations in scale factors and offsets due to the relatively small (2-4°C, see Figures 5 & 6) diurnal variations in temperature. Accuracy estimates indicated by the filter (described below) were on the order of ±2° in roll and pitch and ±4° in yaw.

*Initialization* - The initial roll and pitch angles were estimated from the first accelerometer measurements using equations 2 and 3. The initial yaw angle was estimated by minimizing the sum squared error between the predicted and measured magnetic field strengths given the initial roll and pitch angles.

(2 & 3)
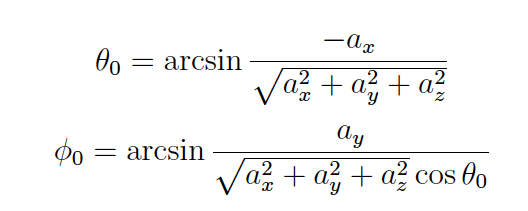


The initial Euler angles were then converted to the initial quaternion estimate with equation 4. The covariance matrix was initialized to *P*_0_ = *diag*( 0.01 0.01 0.01 0.01)

(4)
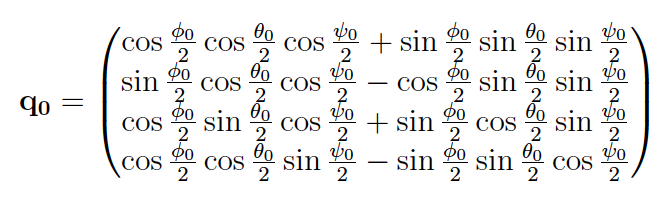


We filtered out those instances where there was a high probability that the sensor was measuring the movement of the egg in addition to gravity. The magnitude of the acceleration was tested to be with 2.5% of 1g. It should be noted that the yaw angle is computed relative to the local magnetic North, leading to the true field strength vector multiplying *T* in the lower half of equation 5. A requirement of attitude quaternions is that they should always be unit norm [44].

(5)
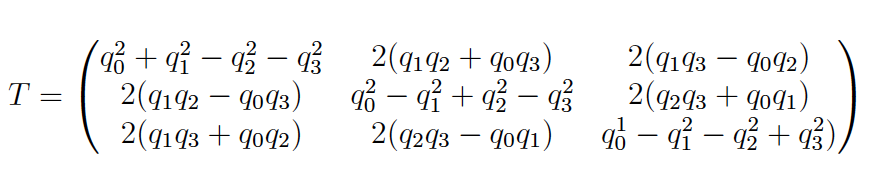


*Estimating Rotation Statistics* - After propagating the attitude state as a quaternion, the more intuitive 3-2-1 Euler angles, often called yaw (Ψ), pitch (θ), and roll (ϕ) were computed using equations 6-8. The Euler angle uncertainties were estimated from the quaternion covariance matrix as P*_Euler_* = J*_Euler2quat_* P*_k_* (J*_Euler2quat_*)^T^ where J*_Euler2quat_* is the Jacobian of the Euler angles with respect to the quaternions.

(6-8)
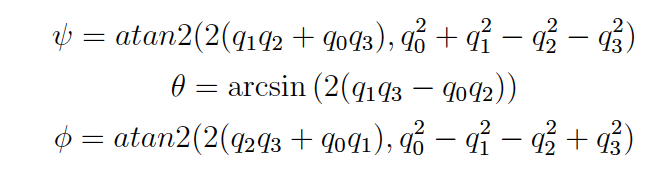


*Data Analysis* – All primary (above) and secondary post-processing of data was performed using custom routines created in MATLAB (The Mathworks, Natick, MA USA). For comparison of behaviors between different species, a number of statistics were computed following the post processing. The main input required for these was to identify egg `turning events' and to quantify how much the egg rotated during each event and how long it took. We applied Euler's rotation theorem between the orientations at each successive time step to determine the axis and minimum angle through which the egg rotated to get from one orientation to the next. This was done by computing the direction cosine matrices corresponding to the orientations at times 1 and 2 (*R_1_* and *R_2_*) using the transpose of equation 5 from above.

The incremental rotation matrix was calculated from these direction cosine matrices *R_inc_* = *R_1_^-1^ R_2_* and the angle of rotation and body frame axis of rotation were calculated using equations 9 and 10.

(9)
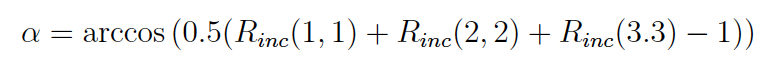


(10)
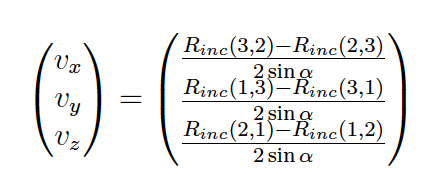


Due to sensor noise, the data were smoothed over a 1-minute window centered about the time of interest (using a 61 point Hamming function). This window was long enough that noise integrated to a small value, but actual rotations show up as significant angle differences. This was in effect a heavily smoothed angular rate signal. We employed a threshold of 0.03 rad/s, which was large enough to exceed the noise floor, but small enough to detect small rotation changes. Rotation events were then identified as consecutive time windows where the threshold was exceeded. For each of these identified events, Euler's theorem was reapplied to find the minimum rotation angle between the initial and final events. For each rotation event, we calculated the minimum rotation angle needed to go from the initial to final orientation, and the start and end times of a rotation.

**References**

41. Astheimer, LB (1986) Egg formation in Cassin’s auklet. Auk 103: 682–693.

42. Maus S, Macmillan S, McLean S, Hamilton B, Thomson A, et al. (2010) The US/UK world magnetic model for 2010-2015.

43. Dorveaux E, Vissière D, Martin A-P, Petit N (2009) Iterative calibration method for inertial and magnetic sensors. Decision and Control, 2009 held jointly with the 2009 28^th^ Chinese Control Conference. CDC/CCC 2009. Proceedings of the 48^th^ IEEE Conference on IEEE.

44 Crassidis JL, Markley FL, Cheng Y (2007) Survey of nonlinear attitude estimation methods. J Guid Control Dynam 30: 12-28.
